# 10a-Hy­droxy-9-(4-meth­oxy­phen­yl)-3,4,5,6,7,8a,9,10a-octa­hydro-1*H*-xanthene-1,8(2*H*)-dione

**DOI:** 10.1107/S160053681203005X

**Published:** 2012-07-07

**Authors:** Hoong-Kun Fun, Chin Wei Ooi, B. Palakshi Reddy, V. Vijayakumar, S. Sarveswari

**Affiliations:** a X-ray Crystallography Unit, School of Physics, Universiti Sains Malaysia, 11800 USM, Penang, Malaysia; b Organic Chemistry Division, School of Advanced Sciences, VIT University, Vellore 632 014, India

## Abstract

In the title compound, C_20_H_22_O_5_, the tetra­hydro­pyran, cyclo­hexene and cyclo­hexane rings of the xanthene ring system adopt half-chair, half-boat and chair conformations, respectively. The mean plane of the four roughly planar atoms of the tetra­hydro­pyran ring (r.m.s. deviation = 0.111 Å) forms a dihedral angle of 82.91 (4)° with the meth­oxy­benzene group. In the crystal, mol­ecules are linked *via* O—H⋯O and C—H⋯O hydrogen bonds into sheets lying parallel to the *ac* plane. The crystal is further consolidated by weak C—H⋯π inter­actions.

## Related literature

For background to the applications of xanthene, see: Menchen *et al.* (2003 ▶); Knight & Stephens (1989 ▶). For our previous studies in this area, see: Palakshi Reddy *et al.* (2010 ▶); Reddy *et al.* (2009 ▶). For ring conformations, see: Cremer & Pople (1975 ▶). For a related structure, see: Loh *et al.* (2011 ▶). For bond length data, see: Allen *et al.* (1987 ▶). For the stability of the temperature controller used for data collection, see: Cosier & Glazer (1986 ▶).
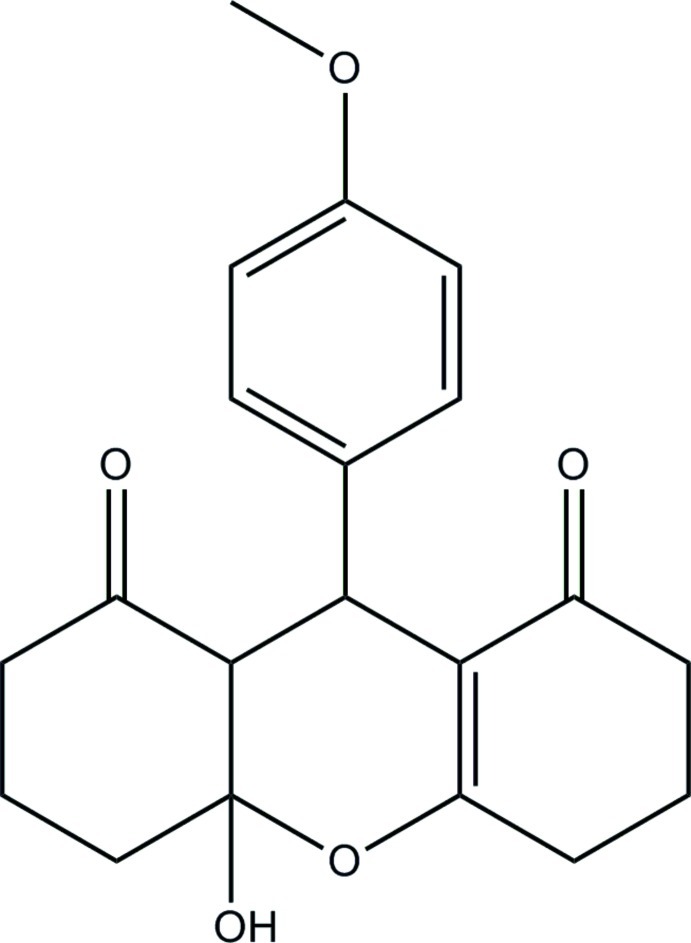


## Experimental

### 

#### Crystal data

C_20_H_22_O_5_
*M* *_r_* = 342.38
Orthorhombic, 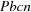

*a* = 15.7611 (9) Å
*b* = 18.0089 (11) Å
*c* = 11.6451 (7) Å
*V* = 3305.3 (3) Å^3^
*Z* = 8
Mo *K*α radiation
μ = 0.10 mm^−1^
*T* = 100 K
0.48 × 0.23 × 0.11 mm

#### Data collection

Bruker APEX DUO CCD diffractometer
Absorption correction: multi-scan (*SADABS*; Bruker, 2009 ▶) *T*_min_ = 0.954, *T*_max_ = 0.990
97212 measured reflections
7190 independent reflections
6068 reflections with *I* > 2σ(*I*)
*R*_int_ = 0.045

#### Refinement

*R*[*F* ^2^ > 2σ(*F* ^2^)] = 0.037
*wR*(*F* ^2^) = 0.108
*S* = 1.05
7190 reflections
231 parameters
H atoms treated by a mixture of independent and constrained refinement
Δρ_max_ = 0.55 e Å^−3^
Δρ_min_ = −0.24 e Å^−3^

### 

Data collection: *APEX2* (Bruker, 2009 ▶); cell refinement: *SAINT* (Bruker, 2009 ▶); data reduction: *SAINT*; program(s) used to solve structure: *SHELXTL* (Sheldrick, 2008 ▶); program(s) used to refine structure: *SHELXTL*; molecular graphics: *SHELXTL*; software used to prepare material for publication: *SHELXTL* and *PLATON* (Spek, 2009 ▶).

## Supplementary Material

Crystal structure: contains datablock(s) global, I. DOI: 10.1107/S160053681203005X/hb6880sup1.cif

Structure factors: contains datablock(s) I. DOI: 10.1107/S160053681203005X/hb6880Isup2.hkl

Supplementary material file. DOI: 10.1107/S160053681203005X/hb6880Isup3.cml

Additional supplementary materials:  crystallographic information; 3D view; checkCIF report

## Figures and Tables

**Table 1:** Hydrogen-bond geometry (Å, °) *Cg*1 is the centroid of the C2–C7 ring.

*D*—H⋯*A*	*D*—H	H⋯*A*	*D*⋯*A*	*D*—H⋯*A*
O4—H1*O*4⋯O3^i^	0.886 (17)	1.935 (17)	2.8156 (8)	172.0 (16)
C12—H12*B*⋯O4^ii^	0.99	2.50	3.1879 (9)	126
C12—H12*A*⋯*Cg*1^iii^	0.99	2.78	3.6557 (8)	147
C16—H16*A*⋯*Cg*1^iv^	0.99	2.78	3.7467 (8)	165

Symmetry codes: (i) 
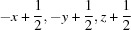
; (ii) 
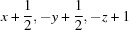
; (iii) 
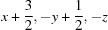
; (iv) 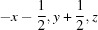.

**Fig. 1.**
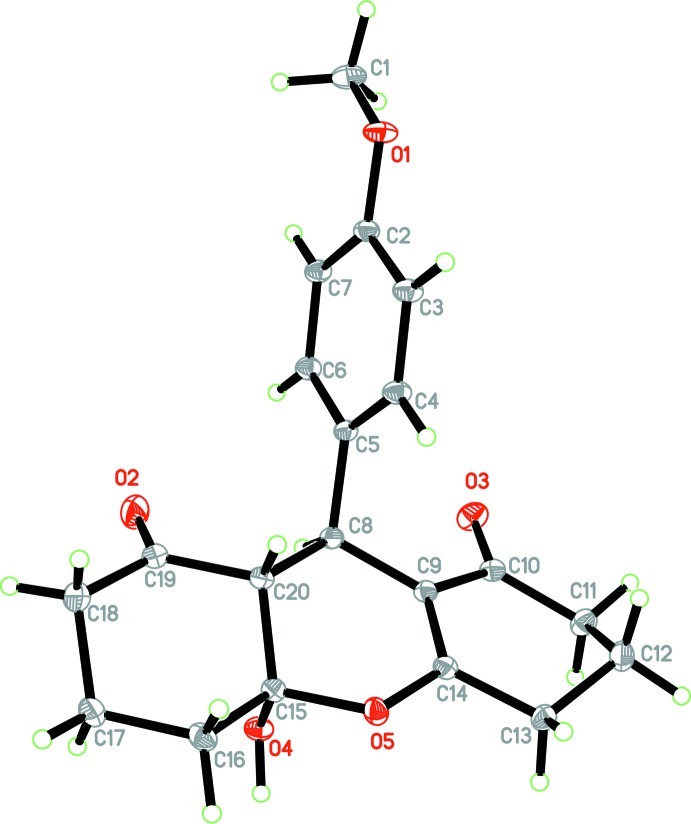
The molecular structure of the title compound, showing 50% probability displacement ellipsoids.

**Fig. 2.**
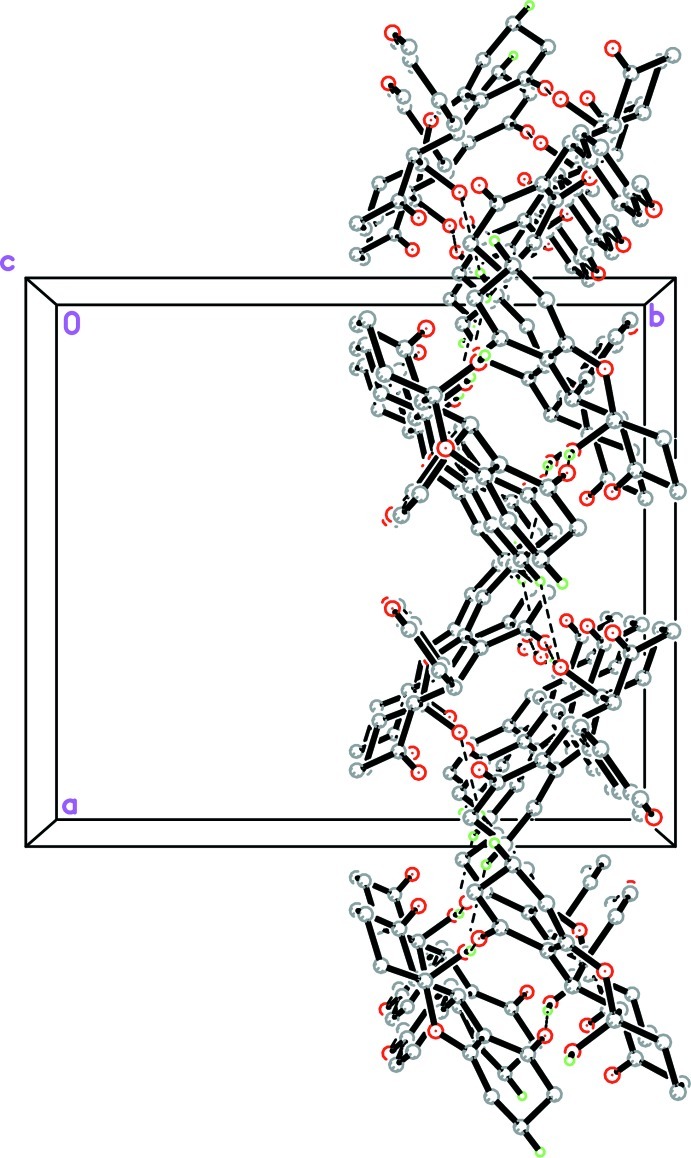
The crystal packing of the title compound, viewed along the c axis. H atoms not involved in the intermolecular interactions (dashed lines) have been omitted for clarity.

## supplementary crystallographic information

### Comment

Xanthene derivatives are important heterocyclic compounds: their uses vary from dyes (Menchen *et al.*, 2003) to agricultural bactericides (Knight *et al.*, 1989). In continuation of our earlier interest in 1,4-DHP's and piperidones (Palakshi Reddy *et al.* 2009; Palakshi Reddy *et al.* 2010), herein we report the crystal structure of the title compound.

In the title compound (Fig. 1), the xanthene ring system consists of three rings which adopt different conformations. The tetrahydropyran ring (O5/C8/C9/C14/C15/C20) adopts a half chair conformation with the puckering parameters Q = 0.4980 (7) Å, θ = 122.73 (8)°, φ = 104.23 (9)° (Cremer & Pople, 1975). The cyclohexene (C9–C14) and cyclohexane (C15–C20) rings adopt half boat and chair conformations with the puckering parameters Q = 0.4905 (8) Å, θ = 117.37 (9)°, φ = 349.74 (10)° and Q = 0.5575 (8) Å, θ = 176.39 (8)°, φ = 192.6 (13)° (Cremer & Pople, 1975), respectively. The mean plane of the tetrahydropyran ring [r.m.s deviation = 0.111 Å] forms a dihedral angle of 82.91 (4)° with the methoxyphenyl group (C1–C7/O1). The bond lengths and angles are comparable to those in a related structure (Loh *et al.*, 2011).

In the crystal structure (Fig. 2), the molecules are linked *via* intermolecular O4—H1O4···O3 and C12—H12B···O4 hydrogen bonds (Table 1) into two-dimensional networks parallel to the *ac* plane. The crystal structure is further consolidated by weak C—H···π interactions (Table 1), involving the centroid of the benzene ring (C2–C7; *Cg*1).

### Experimental

A mixture of 4-methoxybenzaldehyde (1 mol) and 1,3-cyclohexanedione (2 mol) was refluxed in acetonitrile for 3 h. The progress of the reaction was monitored by TLC. After completion of the reaction, it was kept for 2 days for solid formation. The pure product was obtained by recrystallization from acetonitrile in the form of colourless blocks. *M.p.*: 194–196°C; Yield 70%.

### Refinement

Atom H1O4 was located from the difference map and was refined freely [O–H = 0.887 (17) Å]. The remaining H atoms were positioned geometrically and refined using a riding model with *U*_iso_(H) = 1.2 or 1.5*U*_eq_(C) (C–H = 0.95, 0.98, 0.99 and 1.00 Å). A rotating group model was applied to the methyl group. In the final refinement, one outlier (1 0 4) was omitted.

### Crystal data

**Table:**

C_20_H_22_O_5_	*F*(000) = 1456
*M**_r_* = 342.38	*D*_x_ = 1.376 Mg m^−^^3^
Orthorhombic, *P**b**c**n*	Mo *K*α radiation, λ = 0.71073 Å
Hall symbol: -P 2n 2ab	Cell parameters from 9871 reflections
*a* = 15.7611 (9) Å	θ = 2.6–34.8°
*b* = 18.0089 (11) Å	µ = 0.10 mm^−^^1^
*c* = 11.6451 (7) Å	*T* = 100 K
*V* = 3305.3 (3) Å^3^	Block, colourless
*Z* = 8	0.48 × 0.23 × 0.11 mm

### Data collection

**Table:**

Bruker APEX DUO CCD diffractometer	7190 independent reflections
Radiation source: fine-focus sealed tube	6068 reflections with *I* > 2σ(*I*)
Graphite monochromator	*R*_int_ = 0.045
φ and ω scans	θ_max_ = 34.8°, θ_min_ = 1.7°
Absorption correction: multi-scan (*SADABS*; Bruker, 2009)	*h* = −25→25
*T*_min_ = 0.954, *T*_max_ = 0.990	*k* = −28→28
97212 measured reflections	*l* = −18→18

### Refinement

**Table:**

Refinement on *F*^2^	Primary atom site location: structure-invariant direct methods
Least-squares matrix: full	Secondary atom site location: difference Fourier map
*R*[*F*^2^ > 2σ(*F*^2^)] = 0.037	Hydrogen site location: inferred from neighbouring sites
*wR*(*F*^2^) = 0.108	H atoms treated by a mixture of independent and constrained refinement
*S* = 1.05	*w* = 1/[σ^2^(*F*_o_^2^) + (0.059*P*)^2^ + 0.8159*P*] where *P* = (*F*_o_^2^ + 2*F*_c_^2^)/3
7190 reflections	(Δ/σ)_max_ = 0.001
231 parameters	Δρ_max_ = 0.55 e Å^−^^3^
0 restraints	Δρ_min_ = −0.24 e Å^−^^3^

### Special details

**Table:**

Experimental. The crystal was placed in the cold stream of an Oxford Cryosystems Cobra open-flow nitrogen cryostat (Cosier & Glazer, 1986) operating at 100 (1) K.
Geometry. All e.s.d.'s (except the e.s.d. in the dihedral angle between two l.s. planes) are estimated using the full covariance matrix. The cell e.s.d.'s are taken into account individually in the estimation of e.s.d.'s in distances, angles and torsion angles; correlations between e.s.d.'s in cell parameters are only used when they are defined by crystal symmetry. An approximate (isotropic) treatment of cell e.s.d.'s is used for estimating e.s.d.'s involving l.s. planes.
Refinement. Refinement of *F*^2^ against ALL reflections. The weighted *R*-factor *wR* and goodness of fit *S* are based on *F*^2^, conventional *R*-factors *R* are based on *F*, with *F* set to zero for negative *F*^2^. The threshold expression of *F*^2^ > σ(*F*^2^) is used only for calculating *R*-factors(gt) *etc*. and is not relevant to the choice of reflections for refinement. *R*-factors based on *F*^2^ are statistically about twice as large as those based on *F*, and *R*- factors based on ALL data will be even larger.

### Fractional atomic coordinates and isotropic or equivalent isotropic displacement parameters (Å^2^)

**Table:**

	*x*	*y*	*z*	*U*_iso_*/*U*_eq_	
O1	0.41955 (4)	0.43646 (3)	−0.11953 (4)	0.01606 (10)	
O2	0.12092 (4)	0.39264 (4)	0.24042 (6)	0.02378 (13)	
O3	0.35506 (4)	0.19277 (3)	0.24263 (5)	0.01785 (11)	
O4	0.18491 (3)	0.32355 (3)	0.50833 (5)	0.01370 (10)	
O5	0.32107 (3)	0.36949 (3)	0.53699 (4)	0.01306 (10)	
C1	0.38478 (5)	0.41214 (5)	−0.22594 (6)	0.01835 (14)	
H1A	0.4187	0.4321	−0.2894	0.028*	
H1B	0.3262	0.4299	−0.2328	0.028*	
H1C	0.3855	0.3578	−0.2289	0.028*	
C2	0.38251 (4)	0.40829 (4)	−0.02218 (5)	0.01168 (11)	
C3	0.42230 (5)	0.42661 (4)	0.08114 (6)	0.01375 (12)	
H3A	0.4718	0.4567	0.0811	0.016*	
C4	0.38909 (4)	0.40059 (4)	0.18390 (6)	0.01299 (11)	
H4A	0.4165	0.4130	0.2540	0.016*	
C5	0.31611 (4)	0.35644 (4)	0.18628 (5)	0.01067 (11)	
C6	0.27766 (4)	0.33891 (4)	0.08261 (6)	0.01223 (11)	
H6A	0.2279	0.3092	0.0828	0.015*	
C7	0.31024 (4)	0.36386 (4)	−0.02199 (6)	0.01273 (11)	
H7A	0.2834	0.3507	−0.0921	0.015*	
C8	0.27857 (4)	0.33101 (4)	0.30012 (5)	0.01100 (11)	
H8A	0.2310	0.2958	0.2841	0.013*	
C9	0.34387 (4)	0.29187 (4)	0.37349 (5)	0.01071 (11)	
C10	0.37999 (4)	0.22215 (4)	0.33249 (6)	0.01227 (11)	
C11	0.44481 (5)	0.18319 (4)	0.40660 (6)	0.01532 (12)	
H11A	0.4845	0.1553	0.3568	0.018*	
H11B	0.4155	0.1470	0.4568	0.018*	
C12	0.49507 (5)	0.23714 (4)	0.48070 (6)	0.01564 (12)	
H12A	0.5300	0.2698	0.4312	0.019*	
H12B	0.5337	0.2092	0.5320	0.019*	
C13	0.43452 (4)	0.28408 (4)	0.55218 (6)	0.01375 (12)	
H13A	0.4103	0.2532	0.6144	0.016*	
H13B	0.4664	0.3253	0.5882	0.016*	
C14	0.36412 (4)	0.31523 (4)	0.48106 (6)	0.01110 (11)	
C15	0.23657 (4)	0.38542 (4)	0.49304 (6)	0.01137 (11)	
C16	0.20491 (5)	0.45323 (4)	0.55788 (6)	0.01448 (12)	
H16A	0.2459	0.4945	0.5488	0.017*	
H16B	0.2004	0.4415	0.6407	0.017*	
C17	0.11806 (5)	0.47705 (4)	0.51201 (7)	0.01725 (13)	
H17A	0.1003	0.5234	0.5509	0.021*	
H17B	0.0758	0.4382	0.5303	0.021*	
C18	0.11950 (5)	0.48996 (5)	0.38134 (7)	0.02103 (15)	
H18A	0.0610	0.4990	0.3537	0.025*	
H18B	0.1538	0.5346	0.3640	0.025*	
C19	0.15647 (5)	0.42367 (4)	0.31926 (6)	0.01632 (13)	
C20	0.24286 (4)	0.39890 (4)	0.36411 (6)	0.01216 (11)	
H20A	0.2833	0.4409	0.3521	0.015*	
H1O4	0.1773 (11)	0.3167 (9)	0.5830 (15)	0.044 (4)*	

### Atomic displacement parameters (Å^2^)

**Table:**

	*U*^11^	*U*^22^	*U*^33^	*U*^12^	*U*^13^	*U*^23^
O1	0.0198 (2)	0.0200 (2)	0.0083 (2)	−0.00409 (19)	0.00179 (17)	0.00088 (17)
O2	0.0187 (3)	0.0334 (3)	0.0192 (3)	0.0069 (2)	−0.0042 (2)	−0.0018 (2)
O3	0.0242 (3)	0.0162 (2)	0.0131 (2)	0.0013 (2)	−0.00347 (19)	−0.00439 (18)
O4	0.0146 (2)	0.0138 (2)	0.0126 (2)	−0.00304 (16)	0.00192 (16)	0.00182 (17)
O5	0.0119 (2)	0.0149 (2)	0.0124 (2)	0.00174 (16)	−0.00052 (16)	−0.00358 (16)
C1	0.0258 (4)	0.0206 (3)	0.0086 (3)	−0.0017 (3)	0.0007 (2)	−0.0008 (2)
C2	0.0138 (3)	0.0127 (3)	0.0085 (2)	0.0004 (2)	0.00115 (19)	0.00019 (19)
C3	0.0145 (3)	0.0166 (3)	0.0101 (2)	−0.0037 (2)	0.0003 (2)	0.0000 (2)
C4	0.0137 (3)	0.0161 (3)	0.0092 (2)	−0.0031 (2)	−0.0005 (2)	−0.0003 (2)
C5	0.0116 (2)	0.0115 (2)	0.0089 (2)	0.00015 (19)	0.00017 (19)	0.00024 (19)
C6	0.0130 (3)	0.0134 (3)	0.0103 (2)	−0.0016 (2)	−0.00051 (19)	−0.0007 (2)
C7	0.0144 (3)	0.0146 (3)	0.0092 (2)	−0.0012 (2)	−0.0010 (2)	−0.0008 (2)
C8	0.0113 (2)	0.0122 (2)	0.0095 (2)	−0.00026 (19)	0.00045 (19)	0.00068 (19)
C9	0.0119 (2)	0.0110 (2)	0.0093 (2)	0.00007 (19)	0.00030 (19)	−0.00021 (19)
C10	0.0143 (3)	0.0119 (2)	0.0106 (2)	−0.0004 (2)	0.0003 (2)	−0.0001 (2)
C11	0.0182 (3)	0.0133 (3)	0.0145 (3)	0.0032 (2)	−0.0021 (2)	−0.0012 (2)
C12	0.0130 (3)	0.0175 (3)	0.0164 (3)	0.0025 (2)	−0.0017 (2)	−0.0023 (2)
C13	0.0128 (3)	0.0165 (3)	0.0119 (3)	0.0010 (2)	−0.0021 (2)	−0.0016 (2)
C14	0.0110 (2)	0.0116 (2)	0.0107 (2)	−0.00030 (19)	0.00070 (19)	−0.00083 (19)
C15	0.0111 (3)	0.0117 (2)	0.0114 (2)	−0.00005 (19)	0.00047 (19)	−0.0001 (2)
C16	0.0159 (3)	0.0133 (3)	0.0143 (3)	0.0013 (2)	0.0031 (2)	−0.0016 (2)
C17	0.0165 (3)	0.0179 (3)	0.0173 (3)	0.0049 (2)	0.0041 (2)	0.0010 (2)
C18	0.0232 (3)	0.0223 (3)	0.0176 (3)	0.0103 (3)	0.0031 (3)	0.0032 (3)
C19	0.0157 (3)	0.0199 (3)	0.0133 (3)	0.0047 (2)	0.0021 (2)	0.0039 (2)
C20	0.0130 (3)	0.0132 (3)	0.0103 (2)	0.0011 (2)	0.0019 (2)	0.0011 (2)

### Geometric parameters (Å, º)

**Table:**

O1—C2	1.3724 (8)	C9—C14	1.3595 (9)
O1—C1	1.4240 (9)	C9—C10	1.4590 (9)
O2—C19	1.2121 (10)	C10—C11	1.5102 (10)
O3—C10	1.2366 (8)	C11—C12	1.5220 (10)
O4—C15	1.3914 (8)	C11—H11A	0.9900
O4—H1O4	0.887 (17)	C11—H11B	0.9900
O5—C14	1.3561 (8)	C12—C13	1.5225 (10)
O5—C15	1.4552 (8)	C12—H12A	0.9900
C1—H1A	0.9800	C12—H12B	0.9900
C1—H1B	0.9800	C13—C14	1.4940 (10)
C1—H1C	0.9800	C13—H13A	0.9900
C2—C7	1.3919 (10)	C13—H13B	0.9900
C2—C3	1.3963 (9)	C15—C16	1.5201 (10)
C3—C4	1.3876 (9)	C15—C20	1.5241 (9)
C3—H3A	0.9500	C16—C17	1.5306 (11)
C4—C5	1.3986 (9)	C16—H16A	0.9900
C4—H4A	0.9500	C16—H16B	0.9900
C5—C6	1.3872 (9)	C17—C18	1.5395 (11)
C5—C8	1.5223 (9)	C17—H17A	0.9900
C6—C7	1.3961 (9)	C17—H17B	0.9900
C6—H6A	0.9500	C18—C19	1.5124 (11)
C7—H7A	0.9500	C18—H18A	0.9900
C8—C9	1.5120 (9)	C18—H18B	0.9900
C8—C20	1.5383 (9)	C19—C20	1.5250 (10)
C8—H8A	1.0000	C20—H20A	1.0000
			
C2—O1—C1	116.20 (6)	C13—C12—H12A	109.7
C15—O4—H1O4	108.5 (11)	C11—C12—H12B	109.7
C14—O5—C15	115.53 (5)	C13—C12—H12B	109.7
O1—C1—H1A	109.5	H12A—C12—H12B	108.2
O1—C1—H1B	109.5	C14—C13—C12	111.78 (6)
H1A—C1—H1B	109.5	C14—C13—H13A	109.3
O1—C1—H1C	109.5	C12—C13—H13A	109.3
H1A—C1—H1C	109.5	C14—C13—H13B	109.3
H1B—C1—H1C	109.5	C12—C13—H13B	109.3
O1—C2—C7	124.19 (6)	H13A—C13—H13B	107.9
O1—C2—C3	115.68 (6)	O5—C14—C9	123.23 (6)
C7—C2—C3	120.13 (6)	O5—C14—C13	112.08 (6)
C4—C3—C2	119.60 (6)	C9—C14—C13	124.67 (6)
C4—C3—H3A	120.2	O4—C15—O5	109.43 (5)
C2—C3—H3A	120.2	O4—C15—C16	112.80 (6)
C3—C4—C5	121.28 (6)	O5—C15—C16	106.51 (5)
C3—C4—H4A	119.4	O4—C15—C20	106.96 (5)
C5—C4—H4A	119.4	O5—C15—C20	108.57 (5)
C6—C5—C4	118.13 (6)	C16—C15—C20	112.50 (6)
C6—C5—C8	121.31 (6)	C15—C16—C17	110.21 (6)
C4—C5—C8	120.53 (6)	C15—C16—H16A	109.6
C5—C6—C7	121.69 (6)	C17—C16—H16A	109.6
C5—C6—H6A	119.2	C15—C16—H16B	109.6
C7—C6—H6A	119.2	C17—C16—H16B	109.6
C2—C7—C6	119.17 (6)	H16A—C16—H16B	108.1
C2—C7—H7A	120.4	C16—C17—C18	111.97 (6)
C6—C7—H7A	120.4	C16—C17—H17A	109.2
C9—C8—C5	111.58 (5)	C18—C17—H17A	109.2
C9—C8—C20	110.23 (5)	C16—C17—H17B	109.2
C5—C8—C20	108.97 (5)	C18—C17—H17B	109.2
C9—C8—H8A	108.7	H17A—C17—H17B	107.9
C5—C8—H8A	108.7	C19—C18—C17	111.03 (6)
C20—C8—H8A	108.7	C19—C18—H18A	109.4
C14—C9—C10	118.45 (6)	C17—C18—H18A	109.4
C14—C9—C8	122.42 (6)	C19—C18—H18B	109.4
C10—C9—C8	118.81 (6)	C17—C18—H18B	109.4
O3—C10—C9	121.41 (6)	H18A—C18—H18B	108.0
O3—C10—C11	119.98 (6)	O2—C19—C18	123.23 (7)
C9—C10—C11	118.47 (6)	O2—C19—C20	122.49 (7)
C10—C11—C12	112.31 (6)	C18—C19—C20	114.28 (6)
C10—C11—H11A	109.1	C15—C20—C19	109.02 (5)
C12—C11—H11A	109.1	C15—C20—C8	111.99 (5)
C10—C11—H11B	109.1	C19—C20—C8	113.16 (6)
C12—C11—H11B	109.1	C15—C20—H20A	107.5
H11A—C11—H11B	107.9	C19—C20—H20A	107.5
C11—C12—C13	109.76 (6)	C8—C20—H20A	107.5
C11—C12—H12A	109.7		
			
C1—O1—C2—C7	−5.83 (10)	C10—C9—C14—O5	−165.29 (6)
C1—O1—C2—C3	173.89 (6)	C8—C9—C14—O5	8.13 (10)
O1—C2—C3—C4	179.92 (6)	C10—C9—C14—C13	13.68 (10)
C7—C2—C3—C4	−0.35 (11)	C8—C9—C14—C13	−172.89 (6)
C2—C3—C4—C5	−0.24 (11)	C12—C13—C14—O5	−166.48 (6)
C3—C4—C5—C6	0.28 (10)	C12—C13—C14—C9	14.45 (10)
C3—C4—C5—C8	−177.51 (6)	C14—O5—C15—O4	64.53 (7)
C4—C5—C6—C7	0.28 (10)	C14—O5—C15—C16	−173.25 (6)
C8—C5—C6—C7	178.05 (6)	C14—O5—C15—C20	−51.88 (7)
O1—C2—C7—C6	−179.41 (6)	O4—C15—C16—C17	−63.35 (7)
C3—C2—C7—C6	0.89 (10)	O5—C15—C16—C17	176.59 (5)
C5—C6—C7—C2	−0.86 (10)	C20—C15—C16—C17	57.76 (8)
C6—C5—C8—C9	128.25 (7)	C15—C16—C17—C18	−54.64 (8)
C4—C5—C8—C9	−54.04 (8)	C16—C17—C18—C19	51.97 (9)
C6—C5—C8—C20	−109.80 (7)	C17—C18—C19—O2	127.53 (8)
C4—C5—C8—C20	67.91 (8)	C17—C18—C19—C20	−52.57 (9)
C5—C8—C9—C14	122.43 (7)	O4—C15—C20—C19	67.95 (7)
C20—C8—C9—C14	1.21 (9)	O5—C15—C20—C19	−174.06 (5)
C5—C8—C9—C10	−64.17 (8)	C16—C15—C20—C19	−56.44 (7)
C20—C8—C9—C10	174.62 (6)	O4—C15—C20—C8	−58.05 (7)
C14—C9—C10—O3	169.77 (7)	O5—C15—C20—C8	59.94 (7)
C8—C9—C10—O3	−3.90 (10)	C16—C15—C20—C8	177.56 (6)
C14—C9—C10—C11	−6.00 (9)	O2—C19—C20—C15	−125.90 (8)
C8—C9—C10—C11	−179.66 (6)	C18—C19—C20—C15	54.19 (8)
O3—C10—C11—C12	155.40 (7)	O2—C19—C20—C8	−0.58 (10)
C9—C10—C11—C12	−28.78 (9)	C18—C19—C20—C8	179.51 (6)
C10—C11—C12—C13	55.12 (8)	C9—C8—C20—C15	−34.60 (7)
C11—C12—C13—C14	−47.83 (8)	C5—C8—C20—C15	−157.37 (5)
C15—O5—C14—C9	18.89 (9)	C9—C8—C20—C19	−158.31 (6)
C15—O5—C14—C13	−160.19 (6)	C5—C8—C20—C19	78.92 (7)

### Hydrogen-bond geometry (Å, º)

Cg1 is the centroid of the C2–C7 ring.

**Table:**

*D*—H···*A*	*D*—H	H···*A*	*D*···*A*	*D*—H···*A*
O4—H1*O*4···O3^i^	0.886 (17)	1.935 (17)	2.8156 (8)	172.0 (16)
C12—H12*B*···O4^ii^	0.99	2.50	3.1879 (9)	126
C12—H12*A*···*Cg*1^iii^	0.99	2.78	3.6557 (8)	147
C16—H16*A*···*Cg*1^iv^	0.99	2.78	3.7467 (8)	165

Symmetry codes: (i) −*x*+1/2, −*y*+1/2, *z*+1/2; (ii) *x*+1/2, −*y*+1/2, −*z*+1; (iii) *x*+3/2, −*y*+1/2, −*z*; (iv) −*x*−1/2, *y*+1/2, *z*.
